# A comparison of machine learning algorithms for chemical toxicity classification using a simulated multi-scale data model

**DOI:** 10.1186/1471-2105-9-241

**Published:** 2008-05-19

**Authors:** Richard Judson, Fathi Elloumi, R Woodrow Setzer, Zhen Li, Imran Shah

**Affiliations:** 1 National Center for Computational Toxicology, Office of Research and Development, U.S. Environmental Protection Agency, Research Triangle Park, North Carolina 27711, USA; 2 Dept of Biostatistics University of North Carolina, Chapel Hill, 3126 McGavran-Greenberg Hall, CB #7420, Chapel Hill, NC 27599-7420, USA

## Abstract

**Background:**

Bioactivity profiling using high-throughput *in vitro *assays can reduce the cost and time required for toxicological screening of environmental chemicals and can also reduce the need for animal testing. Several public efforts are aimed at discovering patterns or classifiers in high-dimensional bioactivity space that predict tissue, organ or whole animal toxicological endpoints. Supervised machine learning is a powerful approach to discover combinatorial relationships in complex *in vitro/in vivo *datasets. We present a novel model to simulate complex chemical-toxicology data sets and use this model to evaluate the relative performance of different machine learning (ML) methods.

**Results:**

The classification performance of Artificial Neural Networks (ANN), K-Nearest Neighbors (KNN), Linear Discriminant Analysis (LDA), Naïve Bayes (NB), Recursive Partitioning and Regression Trees (RPART), and Support Vector Machines (SVM) in the presence and absence of filter-based feature selection was analyzed using K-way cross-validation testing and independent validation on simulated *in vitro *assay data sets with varying levels of model complexity, number of irrelevant features and measurement noise. While the prediction accuracy of all ML methods decreased as non-causal (irrelevant) features were added, some ML methods performed better than others. In the limit of using a large number of features, ANN and SVM were always in the top performing set of methods while RPART and KNN (k = 5) were always in the poorest performing set. The addition of measurement noise and irrelevant features decreased the classification accuracy of all ML methods, with LDA suffering the greatest performance degradation. LDA performance is especially sensitive to the use of feature selection. Filter-based feature selection generally improved performance, most strikingly for LDA.

**Conclusion:**

We have developed a novel simulation model to evaluate machine learning methods for the analysis of data sets in which in vitro bioassay data is being used to predict in vivo chemical toxicology. From our analysis, we can recommend that several ML methods, most notably SVM and ANN, are good candidates for use in real world applications in this area.

## Background

A daunting challenge faced by environmental regulators in the U.S. and other countries is the requirement that they evaluate the potential toxicity of a large number of unique chemicals that are currently in common use (in the range of 10,000–30,000) but for which little toxicology information is available. The time and cost required for traditional toxicity testing approaches, coupled with the desire to reduce animal use is driving the search for new toxicity prediction methods [1-3]. Several efforts are starting to address this information gap by using relatively inexpensive, high throughput screening approaches in order to link chemical and biological space [1,4-21]. The U.S. EPA is carrying out one such large screening and prioritization experiment, called ToxCast, whose goal is to develop predictive signatures or classifiers that can accurately predict whether a given chemical will or will not cause particular toxicities [4]. This program is investigating a variety of chemically-induced toxicity endpoints including developmental and reproductive toxicity, neurotoxicity and cancer. The initial training set being used comes from a collection of ~300 pesticide active ingredients for which complete rodent toxicology profiles have been compiled. This set of chemicals will be tested in several hundred *in vitro *assays.

The goal of screening and prioritization projects is to discover patterns or signatures in the set of high throughput *in vitro *assays (high throughput screening or HTS, high content screening or HCS, and genomics) that are strongly correlated with tissue, organ or whole animal toxicological endpoints. One begins with chemicals for which toxicology data is available (training chemicals) and develops and validates predictive classification tools. Supervised machine learning (ML) approaches can be used to develop empirical models that accurately classify the toxicological endpoints from large-scale *in vitro *assay data sets. This approach is similar to QSAR (quantitative structure activity relationship), which uses inexpensive calculated chemical descriptors to classify a variety of chemical phenotypes, including toxicity. By analogy, one could use the term QBAR (for quantitative bio-assay/activity relationship) to describe the use of *in vitro *biological assays to predict chemical activity. The QBAR strategy we describe here is also related to biomarker discovery from large-scale -*omic *data that is used to predict on- or off-target pharmacology in drug development, or to discover accurate surrogates for disease state or disease progression.

The QBAR *in vitro *toxicology prioritization approach faces a number of inter-related biological and computational challenges. First, there may be multiple molecular targets and mechanisms by which a chemical can trigger a biological response. Assuming that these alternative biological mechanisms of action are represented in the data, multiple techniques (including ML methods) may be required to discover the underlying relationships between bioassays and endpoint activity. Second, our present understanding of biological mechanisms of toxicity (often referred to as toxicity pathways) is relatively limited, so that one cannot *a priori *determine which of a set of assays will be relevant to a given toxicity phenotype. As a consequence, the relevant features may be missing from the data set and (potentially many) irrelevant features may be included. Here, by relevant features we mean data from assays that measure processes causally linked to the endpoint of interest. By extension, irrelevant features include data from assays not causally linked to the endpoint. The presence of multiple irrelevant assays or features must be effectively managed by ML methods. Third, due to the high cost of performing the required in vivo studies, there are limited numbers of chemicals for which high quality toxicology data is available, and typically only a small fraction of these will clearly demonstrate the toxic effect being studied. The small numbers of examples and unbalanced distribution of positive and negative instance for a toxicological endpoint can limit the ability of ML methods to accurately generalize. In order to develop effective QBAR models of toxicity, these issues must be considered in the ML strategy.

Four critical issues for evaluating the performance of ML methods on complex datasets are: (1) the data set or model; (2) the set of algorithms evaluated; (3) the method that is used to assess the accuracy of the classification algorithm; and (4) the method that is used for feature selection. In order to address the first issue, it was necessary to develop a model of chemical toxicity that captured the key points of the information flow in a biological system. The mathematical model we use is based on the following ideas.

1. There are multiple biological steps connecting the initial interaction of a molecule with its principle target(s) and the emergence of a toxic phenotype. The molecular interaction can trigger molecular pathways, which when activated may lead to the differential activation of more complex cellular processes. Once enough cells are affected, a tissue or organ level phenotype can emerge.

2. There will often be multiple mechanisms that give rise to the same phenotype, and this multiplicity of causal mechanisms likely exists at all levels of biological organization. Multiple molecular interactions can lead to a single pathway being differentially regulated. Up-regulation of multiple pathways can lead to the expression of the same cellular phenotype. This process continues through the levels of tissue, organ and whole animal. One can think of the chain of causation between molecular triggers and endpoints as a many-branched tree, potentially with feedback from higher to lower levels of organization.

3. The number of assays one needs to measure is large, given our relative lack of knowledge of the underlying mechanism linking direct chemical interactions with toxic endpoints.

4. The number of example chemicals for which detailed toxicology information is available is relatively limited due to the high cost of generating the data. In most cases, if a chemical is known to be significantly toxic, further development and testing is halted, so it is unusual to have complete, multi-endpoint toxicity data on molecules that are toxic for any given mode. A corollary is that the number of positive examples for any given toxicity endpoint will be very limited, rarely making up more than 10% of all cases. This will limit the power to find true associations between assays and endpoints. A related issue is that most publicly available data sets that one can use for toxicology modeling are heavily biased toward positive or toxic chemicals, because much less public effort is put into performing extensive studies on chemicals that are negative examples. The ToxCast data set is addressing this selection bias by gathering complete data from a set of chemicals without regard to their ultimate toxicity.

5. The available toxicity endpoint data tends to be categorical rather than quantitative. This is due to the nature of the *in vivo *experiments used to evaluate chemical toxicity. Typically, too few animals are tested under any given condition to pinpoint the lowest effective dose or the rate of phenotypic toxicity at a particular dose. Instead, if a toxic effect is seen at a rate statistically above that seen with a negative control, the chemical will be classified as causing that toxicity.

**Figure 1:**
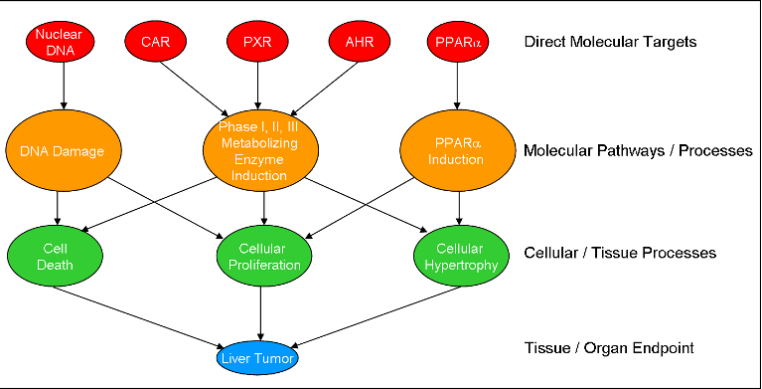
Connections between molecular targets, pathways, cellular processes and endpoints. This is illustrated for 5 molecular targets (nuclear DNA, and the nuclear receptors CAR, PXR, AHR and PPARα), three molecular pathways, and three cellular phenotypes, with liver tumors being the final endpoint. The connections have differing strengths or probabilities and are modulated by the collection of interactions of a given chemical with the molecular targets.

We have developed a simple simulation model which takes into account these ideas. Here we motivate the structure of the model, while the Methods and Results sections provide details. We will illustrate the ideas behind our model with the multiple known pathways that can lead to rodent liver tumors. Several nuclear receptors, including CAR (constitutive androstane receptor), PXR (pregnane-X receptor) and AHR (aryl hydrocarbon receptor), when activated by a xenobiotic, can upregulate a common set of Phase I, Phase II and Phase III metabolizing enzyme pathways [22-24]. Each of these pathways can, when continually activated, lead to cellular phenotypes that include cell proliferation, hypertrophy and cell death. A second, parallel route is activated by compounds that bind to PPARα (peroxisome proliferator-activated receptor α) and lead to cellular hypertrophy and cellular proliferation [24,25]. In a third mechanism, chemicals can directly interact with DNA, causing the activation of DNA damage repair pathways, which can in turn lead to cell death and cellular proliferation. All three of these cellular phenotypes are potential precursors to liver tumors [26]. This collection of interconnected direct molecular targets, target-induced pathways, cellular or tissue phenotypes, and their connections to the endpoint of liver tumors are illustrated in Figure 1.

Our model also assumes that a given chemical can interact with multiple molecular targets. It is well known that many drug compounds interact with multiple targets, as reflected in the phenomenon of off-target toxicity. Relevant to the pathways shown in Figure 1, Moore et al showed that there are compounds that simultaneously activate both CAR and PXR pathways [27]. Preliminary data from the ToxCast program allows us to quantify the magnitude of this multi-target effect. From a set of 183 biochemical targets (primarily receptors, enzymes and ion channels), the 320 ToxCast chemicals[28] (mostly pesticides) were active against an average of 4.2 targets with a maximum of 35, a minimum of 0 and a standard deviation of 5.8.

The connections shown in Figure 1 are not deterministic but instead depend on multiple factors including the strength and duration of the initial chemical-target interaction. Some pathways are more likely than others to lead to the manifestation of particular cellular processes, and some cellular processes are more likely than others to lead to liver tumors. Based on this, one could assign a probability or strength to each arrow in Figure 1. The probability that a given chemical will cause liver tumors is then a complex integral over the individual step-to-step probabilities, modulated by the target interaction strengths for the particular chemical.

There is a vast literature on the evaluation of the performance of different ML methods, but for the present application the literature concerning the analysis of microarray genomics data sets and for QSAR applications are most relevant. Here we describe a pair of representative studies. Ancona et al. [29] used three algorithms (Weighted Voting Algorithm (WVM), Regularized Least Squares (RLS), Support Vector Machine (SVM)) to classify microarray samples as either tumor or normal. They examined the number of training examples that would be required to find a robust classifier. In their example, SVM and RLS outperformed WVM. Statnikov et al. studied all of the major classification issues in the context of multi-category classification using microarray data in cancer diagnosis [30]. They compared multi-category SVM (MC-SVM), k-nearest neighbors (KNN) and several artificial neural network (ANN) implementations and showed that MC-SVM was far superior to the other algorithms they tested in their application.

The literature on machine learning methods in QSAR is equally vast and extends back for 15 years or more. Much of this work (like much of QSAR in general) is focused on the (relatively easy) task of predicting activity against molecular targets. A representative approach to target interaction prediction is the paper by Burbridge et al. comparing SVM to several other algorithms for the prediction of binding to dihydrofolate reductase [31]. Lepp et al performed a similar study that showed SVM performed well in finding predictive QSAR models for a series of 21 molecular targets [32]. The recent state of the science for predicting whole animal toxicity using ML and QSAR methods were reviewed by Helma and Kramer [33], Benigni and Giuliani [34] and by Toivonen et al. [35]. They describe the outcome of an experiment (the Predictive Toxicology Challenge) in which 17 groups submitted 111 models using a training set of 509 NTP compounds for which mouse carcinogenicity data was available. The goal was to predict the carcinogenicity of a set of 185 test compounds. Only 5 of the 111 models performed better than random guessing and the highest positive predictive value for these was 55%, and this model had a false positive rate of 37%. These 5 models[36] include rule-based methods using chemical fragments plus calculated physicochemical properties, a decision tree model, and one using a voting scheme across several standard ML methods. It is difficult to draw many conclusions about the performance of ML methods from this exercise, which failed to produce significantly predictive methods. The authors of these reviews speculate that the cause is a combination of toxicity data being too noisy, the training and test chemical spaces being too large, and structure based approaches being inadequate to predict phenotypes as complex as whole animal toxicity.

One of the key issues in systematically comparing the performance of ML methods is that of estimating accuracy in an unbiased way. For example, Ntzani and Ioannidis [37] report that many of the early studies using microarray data to classify tumor samples did not perform appropriate cross validation, which has led to inflated predictions of classification accuracy. This observation prompted our use of independent validation sets. Molinaro et al. showed that 10-fold cross validation performed well for assessing accuracy of genomics classifiers [38]. Leave one out cross-validation (LOOCV) typically performed somewhat better, but had a significantly higher computational cost. This was assessed by Molinaro et al. in the context of using linear discriminant analysis (LDA), ANN, diagonal discriminant classifiers (DDA), classification and regression trees (CART) and ensemble classifiers. The Molinaro study data set (300 samples and 750 independent variables), which used simulated genomics data, was similar in size to the present work. Baldi, et al. [39] have systematically addressed the issue of ML performance metrics. They describe a number of accuracy metrics including the balanced accuracy or Q-score we use in this paper. The Q-score is the average of the sensitivity and specificity. This is most useful in the case where the classification variable is dichotomous and where the number of positive and negative cases in a training set is not well balanced. They also emphasize that the actual prediction accuracy is related to the similarity of the training and test set.

Finally, Sima and Dougherty examined the issue of finding an optimal subset of features with which to train a classification algorithm [40]. They compare sequential floating forward search (SFFS)[41] and T-test feature selection. This latter can fail when variables are only predictive when they act together. These authors' basic conclusion is that there are optimal subsets of features, but that poor classification performance can be due to either a failure to find an optimal subset or to the inability of any subset to allow accurate classification. This study examined SVM, KNN (n = 3) and LDA as classification algorithms. These authors suggest that automated feature selection methods have inherent limitations and that one should use biologically-based selection when possible. Baker and Kramer used the nearest centroids rule to select small subsets of genes that could be used as robust classifiers from genomics data sets [42]. Kohavi assessed the behavior of cross validation methods to assess classifier accuracy for the C4.5 and Naïve Bayes algorithms [43]. This author concludes that k-fold cross validation with k = 10 provides a good estimate of classification accuracy balanced against modest computational requirements.

In summary, the goal of the analyses we present is to evaluate a machine learning approach to develop classifiers of *in vivo *toxicity using *in vitro *assay data. In order to develop an appropriate ML strategy, we generate simulated QBAR data using a mathematical model whose structure and parameters are motivated by an idealized biological response to chemical exposure based on the following concepts: (a) chemicals interact with multiple molecular targets; (b) exposure to chemicals can stimulate multiple pathways that lead to the same toxicological endpoint; and (c) there are multiple levels of biological organization between the direct molecular interaction and the "apical" endpoint. Additional parameters for generating simulated data include model complexity, the level of noise in the features, the number of chemicals to be screened and the number of irrelevant features. We focus on the special case where there is a large imbalance between the fraction of positive and negative examples, which is found to be the case from our toxicological data [44]. The performance of ML methods is analyzed as a function of these parameters.

## Results

We evaluated the performance of different ML methods on simulated data sets generated by a biologically motivated analytic model. Data sets were simulated based on two levels of complexity; the number of irrelevant assays or input features in the data (data not causally connected with the endpoint being predicted); the number of chemicals or instances; and the presence or absence of measurement noise in the data. In all cases, all of the relevant features (causal for the endpoint being predicted) were included in the data set.

**Figure 2:**
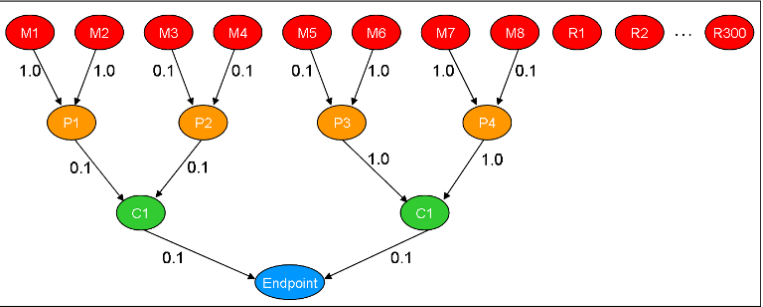
Model S1. The "M" nodes represent assays that measure direct molecular interactions with a chemical. These interactions can activate pathways ("P" nodes) which can in turn activate cellular processes ("C" nodes). Finally, the activation of cellular processes can lead to the presence of an organ or organism-level endpoint. For Model S1, an additional 300 random or "R" nodes were included in the input set of features, so that a total of 308 features are examined. Numerical values shown along edges are values of *w*_*ik *_used in Equation 1.

**Figure 3:**
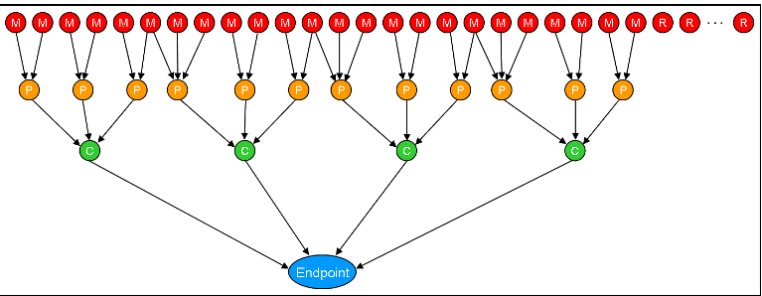
Model S2. All symbols are as described in Figure 1. There are a total of 24 "M" nodes plus 300 "R" nodes for a total of 324 features to be examined.

The network depiction of the simulation models S1 (less complex) and S2 (more complex) are illustrated in Figures 2 and 3. These networks closely resemble the one shown in Figure 1, which models the connections leading from direct molecular interactions with DNA and a variety of nuclear receptors and to liver tumors. Structurally, the simulation models are feed-forward networks that causally link direct molecular interactions (M-nodes) with a final organism-level toxicity endpoint, by way two levels of intervening biological processes. Direct molecular interactions trigger pathway processes (P-nodes) which in turn trigger cellular processes (C nodes). Only if the cellular processes are activated to a sufficient level is the final endpoint manifested. Of equal importance is the fact that many assays will be measured that are not causally linked to the endpoint. These irrelevant nodes are termed R-nodes for random. Our simulations typically include many more R than M nodes or features. Rules for linking molecular interaction strengths to the endpoint are described in the Methods section. The essential points for the present discussion are that a given chemical can interact with one or more input nodes and that the spectrum of input interactions uniquely determines the value of the endpoint.

**Figure 4:**
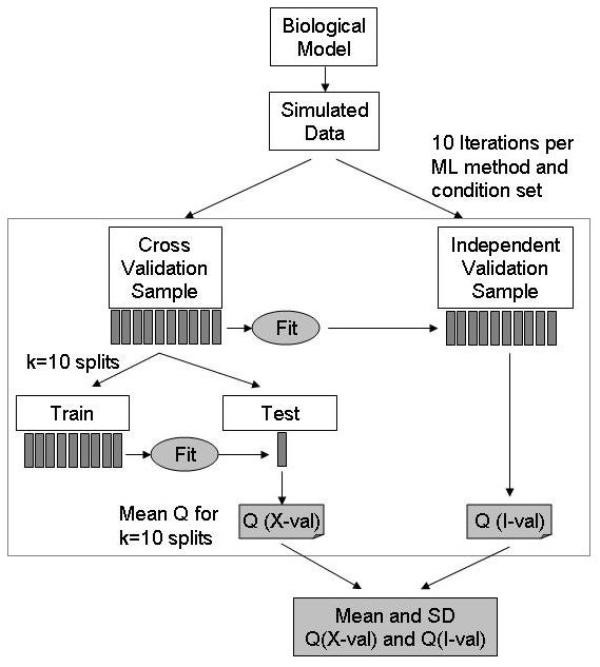
Schematic view of the learning method employed. A large simulated data set is created from the model. From this large data pool, multiple independent samples are drawn and either used for cross validation training and validation (X-val) (left hand branch) or independent model validation (I-val) (right hand branch). For cross validation training, we use standard K-fold cross validation with K = 10. The cross validation performance is the average of the 10 partitions. The classification model ("fit") used in the right hand, independent validation branch is constructed using the entire data set for the left hand branch. For each classifier and each set of conditions, a total of 10 samples are drawn for the cross validation and 10 for the independent validation processes. From this collection of results, we derive means and standard deviations for the balanced accuracy or Q-score.

The performance of LDA (Linear Discriminant Analysis), KNN (k-Nearest Neighbors), SVM (Support Vector Machines), ANN (Artificial Neural Networks), NB (Naïve Bayes) and RPART (Recursive Partitioning and Regression Trees) was evaluated both with and without filter-based feature selection, using 10-way cross-validation testing, as well as validation with independent data sets which included 300 instances. For each set of conditions (ML method, model, number of features, number of chemicals, inclusion of measurement noise, and the presence or absence of filter-based feature selection), training was carried out on 10 independent samples drawn from a simulated data set of 10,000 chemicals. For all evaluations, 10% of the chemicals were positive and 90% were negative for the endpoint being predicted. As mentioned previously, this imbalance between positive and negative examples reflects the situation with the data sets we are modeling in which the adverse phenotypes being studied are rare. Predicted performance was evaluated using K-fold cross-validation with K = 10. For each of the 10 samples, we recorded the number of true positives (TP), false positives (FP), true negatives (TN) and false negatives (FN), sensitivity and specificity and the balanced accuracy or Q-score, which is the average of the sensitivity and specificity. To independently test the performance of the ML method, an independent validation set was drawn from the simulated data set and evaluated with the classification models for each of the 10 training sets. The results (TP, FP, TN, FN, sensitivity, specificity, Q-score) from these 10 data sets were also saved. The approach is outlined in Figure 4.

**Figure 5:**
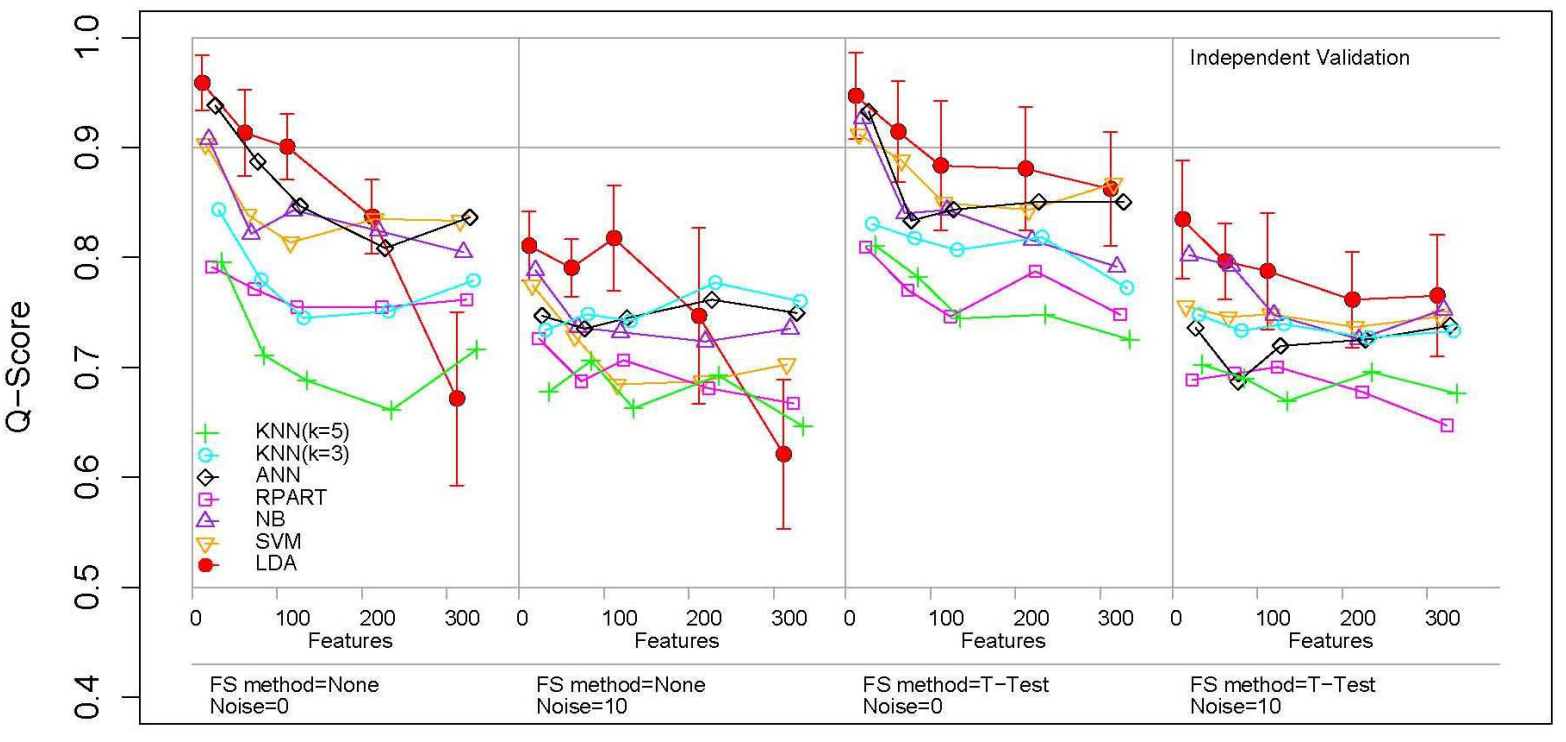
Comparison of ML performance for data set S1. The y-axis shows the Q-Score and the x-axis is divided into four blocks signifying two different conditions: feature selection (none or filter/t-test) and the measurement noise level (0 or 2). In each block the number of input features increases from 8 (all causal for the endpoint) to 308 (300 irrelevant features added). The lines show the performance of different ML methods. Error bars (± 1 SD) are provided for the method LDA to indicate the typical size of variation.

The overall performance results of the different ML methods for the independent validation tests are shown in Figure 5. All results in this figure are calculated using Model S1 (Figure 2) for the case where the training and validation sets contained 300 chemicals or instances. Each panel shows the Q-score trend for the ML methods as a function of the number of features included. Horizontal lines are drawn at Q = 0.9, which is a point that guarantees at least 80% sensitivity and specificity, and at Q = 0.5, which occurs when sensitivity = 0 (all cases are predicted to be negative for the endpoint). The far left point is the case where only the causal features are used. Error bars (± 1 SD) are given for the LDA results to provide an estimate of the level of variation. The other methods showed similar levels of variation. The figure shows the Q-Score curves as a function of increasing number of irrelevant input features in four blocks. In each block, each curve shows the Q-score for one ML method beginning with just the causal features (*N*_*feature *_= 8) and then increasing the number of irrelevant features until *N*_*feature *_= 308. In the first block, the curves generally show a decrease in performance going from *N*_*feature *_= 8 to *N*_*feature *_= 308, which means that the accuracy of all learning methods generally decreased as irrelevant features were added.

The response of different ML methods to the addition of noise varied: LDA and ANN performed the best with only causal features, while with the maximum number of irrelevant features ANN, NB and SVM performed the best and LDA the worst, at least in the absence of feature selection. With the exception of LDA, the performance of different ML methods stabilized after around 100 irrelevant features. With the maximum number of irrelevant features the classification accuracy of KNN and RPART were intermediate between that of the highest group (ANN, SVM, NB) and the lowest (LDA).

The second block from the left shows the classification accuracy of the ML methods without feature selection but with the addition of measurement noise. With no irrelevant features the classification accuracy of all ML methods was significantly lower than in the absence of noise, as expected. LDA showed the same maximum negative performance trend with the addition of irrelevant features. The main difference from the previous case (no noise) was that the performance of KNN (k = 3) was close to that of ANN, NB and SVM as the number of irrelevant features increased. As before, RPART and KNN (k = 5) did not perform well. In general, the classification performance of LDA degraded the most with addition of noise while other methods remained more stable.

The third block from the left shows the classification accuracy of the ML methods with filter-based feature selection (T-test) in the absence of noise. Comparing the performance of the ML methods with the first block (no noise, no feature selection), most ML methods performed better with feature selection but their overall ranking was the same. The exception was LDA, which showed the greatest improvement in performance, tied with SVM and ANN with the greatest Q-score. Feature selection also decreased the overall variability in classification performance between the different ML methods.

The fourth and final block represents the performance results for the ML methods with noise and the use of T-test feature selection. Compared with block 2, where feature selection was not used, the performance of most ML methods increases slightly. LDA showed a significant increase in performance. Compared with block 3, the performance of all techniques was significantly lower when irrelevant features were added. Overall, LDA, NB, SVM, ANN and KNN (N = 3) were quite stable i.e. their performance did not vary tremendously with the addition of noise and irrelevant features.

An alternate way to examine the data is to fix the number of features and look at trends as a function of number of chemicals sampled. These curves (not shown) display the expected trends that as the number of chemicals increases, there is a corresponding improvement in performance. The effects of the variant conditions are basically the same as has already been shown.

Table 1 summarizes the results for both models S1 and S2 for the limiting case where all 300 irrelevant features are included. For all results, 300 chemicals were used. The table is organized into 4 blocks, the same as in Figure 5, but the rows within each block are sorted by decreasing values of Q-score. Values of sensitivity, specificity or Q-score > 0.8 are bolded. Rows shaded in gray have Q-score values less than the best Q-score in that block minus one standard deviation for the best performing method. From this table, one can see that specificity is typically high and that sensitivity is typically low. With a small number of positive cases, a safe classification scheme is to assume that most cases will be negative. The ML methods chiefly differ by their ability to correctly predict the positive cases, which is reflected in the sensitivity. In all cases, KNN (k = 5) and RPART perform poorly relative to the best ML method. In the absence of feature selection, LDA also performs poorly. SVM and ANN are always among the best performers. NB and KNN (k = 3) are intermediate in performance robustness (i.e. relative lack of sensitivity to added noise and number of irrelevant features). The trends for model S2 are not significantly different from those for the simpler model S1. The addition of measurement noise significantly degraded the performance of all ML methods, and this degradation is mainly reflected in poorer sensitivity, i.e. the ability to correctly predict positive cases.

**Table 1:** Performance (mean and SD) of the ML methods.

**Model**	**Learner**	**Noise**	**Feature Selection**	**<Sens>**	**SD(Sens)**	**<Spec>**	**SD(Spec)**	**<Q>**	**SD(Q)**
S1	ANN	0	None	0.71	0.12	**0.96**	0.038	**0.84**	0.068
S1	SVM	0	None	0.68	0.076	**0.99**	0.0063	**0.83**	0.039
S2	SVM	0	None	0.66	0.086	**0.99**	0.0095	**0.82**	0.041
S2	NB	0	None	0.63	0.13	**0.98**	0.0072	**0.81**	0.063
S1	NB	0	None	0.62	0.096	**0.99**	0.0049	**0.8**	0.047
S1	KNN(k = 3)	0	None	0.58	0.13	**0.98**	0.013	0.78	0.067
S2	ANN	0	None	0.56	0.19	**0.98**	0.008	0.77	0.091
S1	CART	0	None	0.56	0.13	**0.96**	0.018	0.76	0.065
S2	KNN(k = 3)	0	None	0.47	0.16	**0.98**	0.014	0.73	0.077
S2	LDA	0	None	0.7	0.11	0.76	0.051	0.73	0.051
S1	KNN(k = 5)	0	None	0.45	0.13	**0.98**	0.014	0.72	0.064
S1	LDA	0	None	0.66	0.13	0.69	0.048	0.67	0.079
S2	KNN(k = 5)	0	None	0.34	0.1	**0.99**	0.016	0.66	0.049
S2	CART	0	None	NA	NA	NA	NA	NA	NA
S1	SVM	0	T-test	0.75	0.089	**0.98**	0.011	**0.87**	0.043
S1	LDA	0	T-test	0.74	0.11	**0.99**	0.0057	**0.86**	0.052
S1	ANN	0	T-test	0.73	0.13	**0.97**	0.023	**0.85**	0.055
S2	LDA	0	T-test	0.67	0.11	**0.98**	0.009	**0.83**	0.054
S2	ANN	0	T-test	0.7	0.11	**0.96**	0.013	**0.83**	0.051
S2	NB	0	T-test	0.65	0.13	**0.98**	0.014	**0.81**	0.064
S2	SVM	0	T-test	0.64	0.11	**0.98**	0.0074	**0.81**	0.057
S1	NB	0	T-test	0.61	0.078	**0.97**	0.012	0.79	0.039
S1	KNN(k = 3)	0	T-test	0.55	0.16	**0.99**	0.008	0.77	0.077
S2	KNN(k = 3)	0	T-test	0.52	0.17	**0.99**	0.0044	0.76	0.082
S2	CART	0	T-test	0.58	0.12	**0.94**	0.025	0.76	0.061
S1	CART	0	T-test	0.54	0.17	**0.96**	0.038	0.75	0.07
S1	KNN(k = 5)	0	T-test	0.45	0.12	**1**	0.0035	0.73	0.062
S2	KNN(k = 5)	0	T-test	0.37	0.13	**0.99**	0.0056	0.68	0.066
S1	KNN(k = 3)	2	None	0.54	0.11	**0.98**	0.011	0.76	0.057
S1	ANN	2	None	0.53	0.13	**0.97**	0.019	0.75	0.066
S1	NB	2	None	0.48	0.13	**0.99**	0.0054	0.74	0.067
S2	KNN(k = 3)	2	None	0.49	0.13	**0.98**	0.0091	0.74	0.065
S2	ANN	2	None	0.44	0.11	**0.98**	0.016	0.71	0.049
S2	NB	2	None	0.43	0.062	**0.99**	0.0056	0.71	0.029
S1	SVM	2	None	0.41	0.09	**1**	0.0031	0.7	0.045
S1	CART	2	None	0.4	0.12	**0.94**	0.041	0.67	0.053
S2	KNN(k = 5)	2	None	0.33	0.087	**0.99**	0.0066	0.66	0.043
S2	CART	2	None	0.34	0.17	**0.96**	0.03	0.65	0.08
S1	KNN(k = 5)	2	None	0.3	0.11	**0.99**	0.0085	0.65	0.054
S2	SVM	2	None	0.3	0.079	**1**	0.0039	0.65	0.039
S2	LDA	2	None	0.59	0.069	0.72	0.05	0.65	0.038
S1	LDA	2	None	0.6	0.12	0.64	0.037	0.62	0.068
S1	LDA	2	T-test	0.55	0.11	**0.98**	0.0078	0.77	0.055
S1	SVM	2	T-test	0.5	0.11	**0.99**	0.004	0.75	0.053
S2	NB	2	T-test	0.53	0.084	**0.98**	0.01	0.75	0.042
S1	NB	2	T-test	0.52	0.083	**0.98**	0.0088	0.75	0.042
S2	LDA	2	T-test	0.52	0.087	**0.97**	0.011	0.75	0.042
S2	SVM	2	T-test	0.48	0.1	**0.99**	0.0056	0.74	0.052
S1	ANN	2	T-test	0.51	0.1	**0.97**	0.015	0.74	0.045
S2	KNN(k = 3)	2	T-test	0.48	0.14	**0.99**	0.0083	0.73	0.07
S1	KNN(k = 3)	2	T-test	0.48	0.1	**0.98**	0.01	0.73	0.051
S2	ANN	2	T-test	0.48	0.1	**0.96**	0.01	0.72	0.049
S1	KNN(k = 5)	2	T-test	0.36	0.095	**1**	0.0049	0.68	0.047
S2	KNN(k = 5)	2	T-test	0.32	0.047	**0.99**	0.0036	0.66	0.023
S1	CART	2	T-test	0.35	0.14	**0.95**	0.023	0.65	0.071
S2	CART	2	T-test	0.25	0.093	**0.96**	0.027	0.6	0.044

This data is compiled for the special case where 300 chemicals were used, as a function of model, feature selection and level of measurement noise. The results are organized into 4 blocks, corresponding to the 4 blocks in Figure 5. Within a block, rows are ordered by decreasing values of Q-Score. The results give the average sensitivity, specificity and Q-score along with their corresponding standard deviations. All ML methods were trained using 300 chemicals. The values come from 10 independent validation runs with unique samples of 300 chemicals. Values of sensitivity, specificity and Q-score > 0.8 are bolded. Rows where the Q-score is less than that of the best Q-score in the block minus one standard deviation for the best row are shaded.

## Discussion

Developing predictive classifiers for complex biological data sets is a challenging problem because there are generally more features than instances (curse of dimensionality); the classification variable and input features are noisy; and there are many irrelevant features (i.e. ones that are measured but which have no causal connection to the value of the classification variable). We have developed a test bed for representing biologically motivated models and have used it to provide insight into the relative classification performance of different ML methods. Though true *in vitro *biological systems are more complex and dynamic than our model, our approach provides empirical insight into the relative performance of different learning methods as a function of the absence and presence of experimental noise and the number of features. In particular, we have focused on the situation which is common in toxicology data sets, namely where there is an imbalance between the number of positive and negative examples.

We find several main trends from our simulated data by systematically analyzing different ML methods on the same testing, training and validation data. First, most ML methods perform well in the presence of a small number of causal features, but most show significant degradation in performance as irrelevant features are added, which is well-known [45]. Second, all ML methods perform better with filter-based feature selection as irrelevant features are added. Third, the performance depends upon noise in the input features. While most ML methods perform well in the absence of noise, some are more stable than others. Fourth, in the presence of noisy and irrelevant features, and with feature selection, most ML methods perform similarly, with the exceptions of RPART and KNN (k = 5) which performed significantly worse. The models (Figures 2 and 3) resemble generalized artificial neural networks, leading one to suspect that ANN methods should perform well. In general this is true, although (see Figure 5) other methods always performed at least as well.

We found that the accuracy predicted using k-fold cross validation was statistically indistinguishable from that seen with an independent validation set except in the case of KNN (k = 3 or 5) with no feature selection. In this case, the k-fold cross validation predicted a higher accuracy than was seen with independent validation. This is the only situation where we detected over-fitting using the training data. This phenomenon disappeared when we tested KNN against a more balanced data set in which there were equal numbers of positive and negative examples. All other parameters were unchanged. Issues arising from unbalanced data sets have been previously analyzed. Japkowicz et al. found that classifier performance of imbalanced datasets depends on the degree of class imbalance, the complexity of the data, the overall size of the training set and the classifier involved [46]. Sun et al. also observed that given a fixed degree of imbalance, the sample size plays a crucial role in determining the "goodness" of a classification model [47]. The KNN method is sensitive to imbalanced training data [48,49], and the class distribution of our simulation data is highly skewed with the positive to negative rate of 1:9, thus the sample size very likely explains the different performance between the training and validation sets.

**Figure 6:**
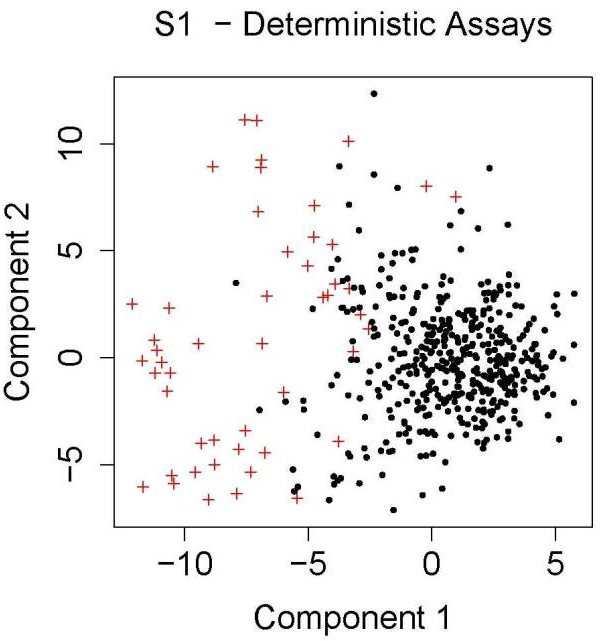
Distribution of chemicals in feature/assay space for model and S2. The data is projected into 2 dimensions using multi-dimensional scaling. Chemicals that are negative for the endpoint are indicated by black circles, and chemicals positive for the endpoint are represented by red crosses. For ease of visualization only a randomly selected set of 500 chemicals are shown.

**Figure 7:**
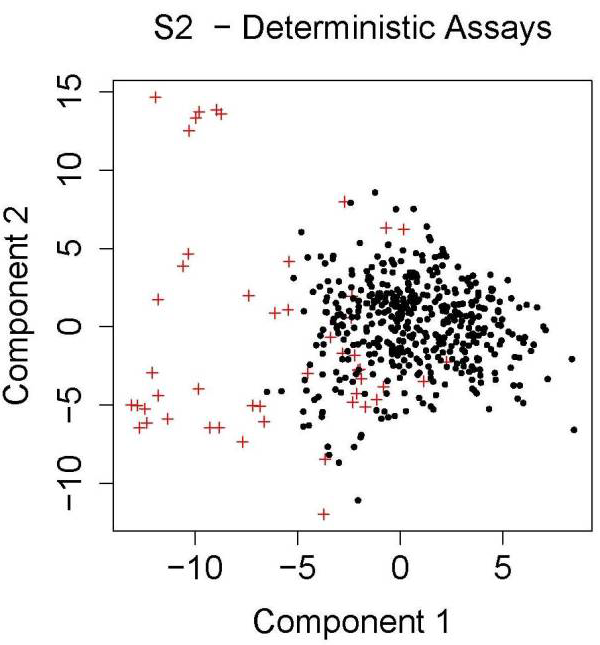
Same as Figure 7, except for model S2.

One of the important limitations of this work is that the performance of classifiers is biased by our model of chemical-induced bioactivity and toxicity. We assume a static deterministic model of a biological system without feedback. An important aspect of the chemical simulation model is the use of multiple chemical classes, each of which contains a collection of chemicals that behave similarly (as measured by their molecular interaction spectrum). As described in the methods section, a chemical class is defined by first creating an example of the class by randomly drawing values for the assay values from a gamma distribution. The other members of the class are created through the addition of normally distributed random values to each assay value of the chemical class exemplar. This process creates a set of (possibly overlapping) clusters in the high dimensional feature space. We then draw samples of chemicals from a wide space in which some clusters contain no chemicals that are positive for the endpoint, some contain only positive cases, and some clusters contain a mix of positive and negative cases. One can qualitatively see how this clustering of feature space will affect the difficulty of the classification problem by projecting the chemical space into 2 dimensions and examining how positive and negative cases cluster. Figures 6 and 7 shows these projections for model S1 (first panel) and S2 (second panel) using only the causal feature values for the distance calculation. Projection was performed using multidimensional scaling (R routine cmdscale). PCA gives similar results. For model S1 the problem is approximately separable, so almost any method should perform well. When all features are included (not shown), there is more mixing, but still the positive cases tend to cluster together, so cluster identity (regardless of whether the causal or the irrelevant features are being used) should be a good surrogate for classification. In model S2 there is still a reasonable separation for the case when only the causal features are included. The presence of a few outlying, all-positive clusters is very obvious when all features are included.

We have focused on the performance of single classifiers, but voting methods which combine the predictions of multiple individual methods have been used. Statnikov et al. studied ensemble classifiers or voting schemes, which attempt to combine multiple sub-optimal classifiers to improve overall accuracy. That paper evaluated the utility of selecting very small subsets of genes (as few as 25 out of > 15,000) for classification. This has the effect of greatly reducing the danger of over-fitting from small numbers of samples. Additionally, these authors demonstrated how to evaluate the comparative performance of different algorithms using permutation testing. Two conclusions from the Statnikov et al. work on cancer diagnosis using microarray data are relevant to the present study. First, they observe that SVM methods outperformed KNN and ANN. Our findings show that the relative rankings of these 3 methods is a complex function of the number of irrelevant features, the level of noise and the use (or not) of feature selection. Second, the authors observed that the ensemble classification methods tended to do worse than single methods. Although we did not evaluate the performance of ensemble based classification, our results (Table 1 or Figure 5) do not suggest that voting would lead to a decrease in performance, as long as the voting rule was that the chemical was labeled positive if any method predicted it to be positive.

The present work limited the number of chemicals to 300 and features to 300, which corresponds to the number of chemicals and assays we are using in the first phase of the ToxCast program. Despite the relatively small size of the data set, we were able to evaluate key issues in supervised learning from noisy and irrelevant data. We plan to expand the number of features and instance in future work as we gain additional insights from experimental data. Additionally, we intend to more fully explore the use of dimensionality reduction (e.g. through correlation analysis of closely related features), feature selection and classifier ensembles in future work.

## Conclusion

The prediction of chemical toxicity is a significant challenge in both the environmental and drug development arenas. Gold standard *in vivo *toxicology experiments in rodents and other species are very expensive and often do not directly provide mechanism of action information. The alternative, which has been widely pursued in the pharmaceutical industry, is to screen compounds using use *in vitro *or cell based assays and to use the results of these assays to prioritize compounds for further efficacy and safety testing. These *in vitro *screening techniques are now being introduced in a significant way into the world of environmental chemical safety assessment. Here, there are unique challenges due to the modest amount of *in vivo *toxicology data that can be used to develop screening models, and due to the broad chemical space covered by environmental chemicals whose toxicology is poorly characterized. The EPA is carrying out a significant screening and prioritization program called ToxCast, whose eventual aim is to screen a large fraction of the commonly used environmental chemicals and to prioritize a subset of these for more detailed testing. The present analysis provides a novel simulation model of the linkage between direct chemical-target interactions and toxicity endpoints, and uses this model to develop guidelines for using ML algorithms to discover significant associations between *in vitro *screening data and *in vivo *toxicology.

We find several main trends from our simulated data set by systematically analyzing different ML methods on the same testing, training and validation data. First, most ML methods perform well in the presence of a small number of causal features, but most show significant degradation in performance as irrelevant features are added, which is well-known [45]. Second, all ML methods perform better with filter-based feature selection as irrelevant features are added. Third, while most ML methods perform well in the absence of measurement noise, some are more stable than others. Fourth, in the presence of noisy and irrelevant features, and with feature selection, most ML methods perform similarly well, with the main exceptions being RPART and KNN which underperformed the other methods.

## Methods

### Simulation Models

**Figure 8:**
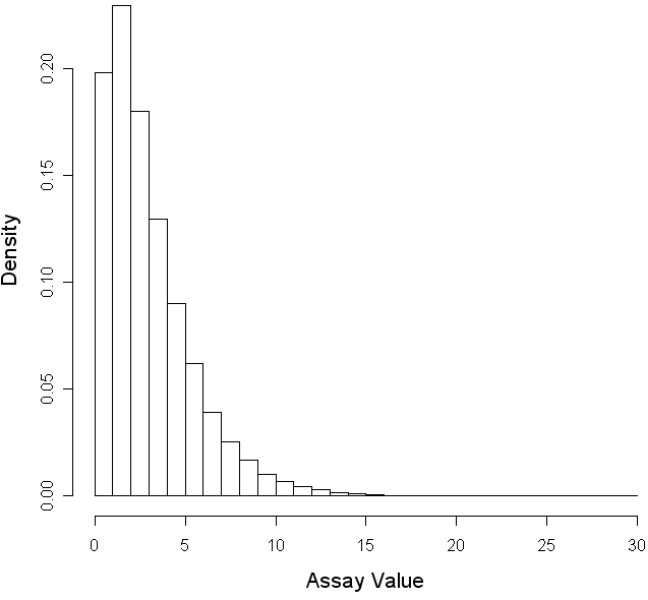
Distribution from which the M and R assay values are drawn. This is a gamma distribution with shape = 3/2 and rate = 0.5

We use two models of the networks connecting direct molecular interactions with a test chemical and the presence or absence of a toxic endpoint. Direct molecular interactions determine values of the M assays in the models. These interactions can trigger pathway processes (P-nodes), which can in turn trigger cellular events (C-nodes), which can finally lead to the expression of a toxic endpoint. In addition to the M nodes, there are a large and variable number of random or R nodes with which a chemical can interact. Throughout the paper, we refer to the M and R nodes as causal and irrelevant node or features, respectively. A simulated chemical is uniquely characterized by its spectrum of activity for the direct molecular interaction assays (M + R nodes). The value of the i-th M (or R) assay for chemical *c *is given by *M*_*i*_(*c*) and is randomly generated from a gamma distribution (shape = 3/2, rate = 0.5, ~95% of values are between 0 and 8). This is the type of distribution one could see for -log(*k*) where *k *is a binding or inhibition constant for a molecule interacting with protein target Figure 8 shows the distribution of values for the M and R assays or features.

The model guarantees that if two molecules have the same spectrum of direct physical interactions, they will exhibit the same downstream biology, including whether or not they cause the endpoint to be activated. By altering the interaction strength connecting nodes in the model, one can simulate differing degrees of coupling between multiple molecular targets and the downstream processes they control.

These networks simulate the ability for an endpoint to be triggered by multiple independent mechanisms. In model S1, there are 2 major mechanisms, driven by the independent cellular processes C1 and C2 (see Figure 2). A collection of chemicals may contain some substances that trigger the endpoint through one mechanism and some through the other. Some chemicals may trigger both. This interplay of multiple mechanisms is characteristic of many toxicological and disease processes and will allow us to evaluate the ability of classification algorithms to identify multiple paths from input to output in a biological system.

For all P and C nodes, values are calculated using weights for the edges leading into a node plus the values of the parents:

(1) $$X_{i}^{L+1}(c)=\sum_{j}w_{ij}X_{j}^{L}(c)\;+\;\sum_{jk}\frac{1}{2}w_{ijk}\sqrt{X_{j}^{L}(c)\;X_{k}^{L}(c)}\;$$

where $X_{i}^{L}(c)$ is the value for node $N_{i}^{L}(c)$ in level L ∈ [M, R, P, C, Endpoint] for chemical *c*, and *w*_*ik *_and *w*_*ijk *_are weights for the linear and quadratic interaction terms. The quadratic term in Equation 1 simulates the presence of cooperativity between upstream processes that is necessary to trigger downstream processes. In order to test binary classification algorithms, we assign chemicals to the positive (1) class if the value of *X*_*i*_(*c*) for the endpoint node is in the top 2% of the distribution, and to the negative (0) class otherwise. The weights values *w*_*ik *_and *w*_*ijk *_are either 1.0 or 0.1 and are assigned sequentially through the network using the repeating series (1.0, 1.0, 0.1, 0.1, 0.1). For the simulations, 2 different model networks were used, called S1 and S2. The networks are shown in Figures 2 and 3. Model S1 has 2 parents for each node. Model S2 has 4 C-level parents of the endpoint, 3 P-level parents for each C node and 2 M-level parents for each P node. Note that for S2, certain M-level molecular interactions can trigger more than one of the major mechanisms. Figure 2 displays the values of the weights used for the linear portion of the model. Both networks contained a total of 400 input layer nodes or molecular assays (S1: 8 M+392 R; S2: 24 M+374 R), although the simulations only made use of up to 300 R nodes.

### Simulation Data Sets

For each model (S1 and S2), a set of 100,000 chemicals was created with 2% being assigned to the positive endpoint class. The chemicals are not generated completely randomly, but were instead created from 500 chemical classes, each with 200 examples. To create a class, a first example was randomly generated (M and R assays drawn from the gamma distribution) and then the other examples are created from the exemplar by randomly adding normally distributed variation (SD = 1) to each M and R assay. The chemical class value (1...500) was retained with each chemical. From this large set of chemicals, a sampling population was created by drawing 10,000 chemicals from the larger set, but enriching the fraction of positive cases to 10%. This represents a very broad universe of chemicals.

From the set of 10,000 chemicals, multiple samples were drawn and used in the classification training and testing process. The only data given to the classification algorithms are the values for the M and R assays or features and the endpoint classification. A sample was characterized by the following variables:

1. Model (S1, S2)

2. The number of chemicals (50,100,200,300)

3. The number of random or irrelevant features (R nodes) (50,100,200,300)

4. Whether or not measurement noise was added to the original M and R assay values. If so, normally distributed noise (SD = 2) was added to each assay's value.

### Classification Methodology

Each classification algorithm or ML method was evaluated using the balanced accuracy or Q-score [39], which is the average of the sensitivity and specificity for prediction. This is a useful metric in the present situation because the fraction of positive cases is small and the Q-score gives equal weight to the accuracy of predicting positive and negative cases. In each sample, the fraction of chemicals that is positive for the endpoint is small (10%), so a good first approximation would be to predict that all chemicals will be negative for the endpoint. The Q-score for this default prediction is 0.5, whereas a perfect prediction will score 1.0.

Each ML method was evaluated against a set of 10 samples or training sets, each using k-wise cross validation, with k = 10 [43]. The model that was produced from each of the training samples was evaluated against a separate validation sample. The training and validation samples were drawn from the same distribution. We calculated distributions of Q-score for both the training samples (the results of the k-fold cross validation) and the validation samples. We call these the "predicted" and "true" Q-scores.

For each sample set described above, we evaluated performance for a series of ML methods with no feature selection and with T-test filter feature selection. In the latter case, the best 20% of features were selected, with a minimum number of 8. (Note that the features (M-nodes) are not strictly normally distributed, but are instead drawn from a gamma distribution overlaid with normally distributed variation.) To manage the large number of individual runs, a simple MySQL database was created with 2 tables called *queue *and *result*. The queue table contains all run parameters and the result table holds all of the relevant results. The relevant parameters in the queue table are [model (S1, S2), measurement noise (0/2), number of features, number of chemicals, ML method, feature selection mode (none or T-test)]. In all cases, the fraction of positive cases in the sample was 10%. Figure 4 illustrates the overall approach.

### Classification Algorithms/ML methods

Table 2 lists the ML methods that were evaluated, along with any non-default parameters. Parameters for each of the machine learning methods were tuned so that the performance (Q score) was acceptable (> 0.9) when tested against model S1 when the ML method was presented with all of the true features, no irrelevant features, and when no noise was added to the features. Default parameters were used for KNN, NB, LDA and RPART. For SVM, the cost function was varied over the range from 1 to 1000 and a value of 100 was selected. ANN was the only method requiring significant tuning. Approximately 20 combinations of the parameters listed in Table 1 were tested prior to arriving at an acceptable set. All code was written in R (version 2.5.1) using the MLInterfaces implementation of all ML methods. The code was parallelized using snow and Rmpi and run on a Linux workstation cluster and an SGI Altix 4700.

**Table 2:** Classification or ML methods used, along with reference to the R library used.

**ML Method**	**Description**	**Library**
KNN	K-nearest neighbors (N = 3,5)	MLInterfaces [50]
NB	Naïve Bayes	e1071 [51]
LDA	Linear Discriminant Analysis	MLInterfaces [50]
SVM	Support Vector Machine (kernel = radial, cost = 100)	e1071 [51]
ANN	Artificial Neural Networks (size = 10, range = 0.5, decay = 0.0001, maxit = 200, MaxNWts = 10000)	e1071 [51]
RPART	Recursive Partitioning and Regression Trees (method = class, cp = 0, usesurrogate = 2	e1071 [51]

## List of abbreviations

The following abbreviations are used in the manuscript: ML: Machine Learning; KNN: k-Nearest Neighbors; NB: Naïve Bayes; LDA: Linear Discriminant Analysis; SVM: Support Vector Machine; ANN: Artificial Neural Network; CART: Classification and Regression Trees; RPART: Recursive Partitioning and Regression Trees; HTS: High Throughput Screening; HCS: High Content Screening.

## Authors' contributions

RJ, IS, WS, ZL, FE participated in the design of the experiment, in the design of the analysis strategy, in the formulation of the conclusions and in implementation of the analysis software. In addition, RJ developed the simulation model and its software implementation and performed the analysis runs. IS developed the bulk of the final analysis software. RJ and IS drafted the manuscript. All authors read and approved the final manuscript
